# Determinants of hypertension among diabetic patients in Public Hospitals of the Central Zone, Tigray, Ethiopia 2018: unmatched case-control study

**DOI:** 10.11604/pamj.2019.33.100.17094

**Published:** 2019-06-11

**Authors:** Teklewoini Mariye, Alem Girmay, Hagos Tasew, Girmay Teklay, Ebud Ayele, Hadgu Gerensea, Hussen Mokonnen

**Affiliations:** 1 Department of Adult Health Nursing, Aksum University, Tigray, Ethiopia; 2 Department of Pediatric Nursing, Aksum University, Tigray, Ethiopia; 3 Department of Nutrition, Aksum University, Tigray, Ethiopia; 4 Department of Nursing and Midwifery, College of Health Science, Addis Ababa University, Addis Ababa, Ethiopia

**Keywords:** Determinants, diabetic, hypertension, Ethiopia

## Abstract

**Introduction:**

Hypertension, among diabetic patients, is a worldwide public-health challenge and a leading modifiable risk factor for other cardiovascular diseases. The main purpose of this study was to identify determinants of hypertension among diabetic patients.

**Methods:**

Data were collected from January to March 2018 using an interviewer-administered structured questionnaire. Data collectors and supervisors were trained before the period of data collection. The questionnaire was pretested on 5% of the sample at Suhul hospital. Bivariable logistic regression was employed to examine the crude associations between the outcome variable and determinant variables. This was followed by multivariable analysis to examine the determinants of hypertension among diabetic patients by selecting variables which had p value ≤0.2 in the bivariable analysis.

**Results:**

The age range of the respondents was 18-80 years, with the median age of 51.56±14.92 years. Not attending diabetes mellitus education sessions (AOR=2.61, 95% CI (1.12,6.1), duration since diagnosis with diabetes (AOR=8.52; 95% CI (1.97, 36.84), poor glycemic control (AOR=22.99, 95CI (5.92,89.28), overweight (AOR=4.84, 95%CI (1.42,16.51), and non-adherence to diabetes medication (AOR=4.66, 95% CI (2.22,9.79), diet (AOR=9.70,95% CI (3.34,28.22), exercise (AOR= 5.47, 95% CI (2.35,12.75), and self-monitoring blood glucose (AOR=6.62, 95% CI (3.16, 13.86) were found to be the determinants of hypertension among diabetic patients.

**Conclusion:**

This research concludes that longer duration with diabetes, nonattendance of diabetes education sessions, poor glycemic control, and not-adherence to antidiabetic medications, diet, exercise and self-monitoring blood glucose were found to be the determinants of hypertension among diabetic patients.

## Introduction

Hypertension, among diabetic patients, is a worldwide public-health challenge and a leading modifiable risk factor for other cardiovascular diseases and death [1]. The frequency of hypertension among the diabetic population is almost twice of the non-diabetic patients [2]. Besides, compared with other cardiovascular disorders, hypertension is the most common comorbid disease in diabetic patients [3]. The coexistence of hypertension and diabetes mellitus is a major contributor to the development and progression of microvascular and macro vascular complications in people with diabetes mellitus [4]. In Africa, hypertension, which was once a relative rarity, has now changed to a major public health problem [5]. In people with diabetes mellitus, hypertension has become the commonest cause of cardiovascular diseases compared to renal, stroke and other diseases in the continent. If continued with the current trajectory, by 2020, three-fourth of all deaths in Africa will be attributable to hypertension [6]. Hypertension along with diabetes mellitus is a strong cause of vascular complications and the leading cause of morbidity and mortality [7-9]. The coexistence of hypertension in diabetic mellitus is attributed to the risk of death and cardiovascular events by 44% and 41%, respectively, as compared to 7% and 9% of the these risks in people with diabetes alone [7]. Besides, hypertension is also the largest contributor to the direct and indirect costs of the general population [9]. Identifying the determinants of hypertension among diabetic patients will enable healthcare professionals to successfully tackle its impact on patients. Moreover, it may also help health policy-makers in designing or redesigning an appropriate strategy to reduce health associated costs. To date, there is no established evidence regarding the determinants of hypertension among diabetic patients in Ethiopia. Hence, this study was aimed at assessing the determinants of hypertension among diabetic patients in public hospitals of Central Zone of Tigray, Ethiopia.

## Methods

This study was conducted in public Hospitals of Central Zone, Tigray, Ethiopia. The data for this study were collected from January one through March 30, 2018 G.C. Unmatched case-control study design was employed with controls being all diabetic mellitus patients without hypertension and the cases being all diabetic mellitus patients diagnosed with hypertension. Diabetes mellitus patients who were critically ill were excluded from the study. The sample size was calculated by using EPI Info software version 7.1.1 with the following parameters: significance = 95%; power = 80%; Odds ratio = 2.46. The Odds ratio was taken from family history of controls from a study conducted in Durame Town, Southern Ethiopia in 2014 [10]. Case to control ratio was 1:2; proportion of controls with exposure was 66.7%. The proportion of cases with exposure was 83.1%. Assuming a non-response rate of 10%, the sample size for cases and controls were found to be 102 and 204, respectively, which gave us a total sample of 303. A systematic random sampling technique was used to select the study subjects. Per each hospital, Two Ks, one for cases and one for controls, were calculated by dividing the number of cases and controls of the population (N) to their respective number of cases and controls of the sample (n). The subjects were selected every K interval of cases and controls, and the first study subjects were selected by lottery method. The dependent variable for this study was diabetes with hypertension, and the independent variables were socio-demographic factors (sex, age, education status, residence, marital status, occupation, ethnicity and religion), health profiles (BMI status, duration since diagnosed with DM, and other comorbidities) and behavioral factors (adherence to diabetic diet, adherence to exercise, smoking, adherence to diabetic medication and adherence to blood glucose measurement as well as glycemic control). Review of the diabetic patients' record was conducted to identify cases and controls by using checklists. Cases and controls were recorded by identification number. Following segregation of cases and controls, data were collected from record review card and by interviewing the study participants. Beside the checklist, a structured questionnaire was used for data collection. The questionnaire had three parts: part I, social demographic data; part II, clinical characteristics of the study subjects; and part III, behavioral factors. The summary of diabetic care activity (SDCA) was used to measure the behavioral factors such as adherence to exercise, adherence to self-monitoring of blood glucose level, cigarette smoking and alcohol drinking. Moreover, MMS (Modified Morse scale) was used to measure other behavioral factors such as adherence to diabetic medication and diet. The reliability and validity of the SDCA and Modified Morse scale questionnaire were already tested among similar study subjects in other studies conducted in Ethiopia [11].

Their usage in evaluating adherence to medication and diet among diabetic patients was proven to be reliable in similar studies in our country [12, 13]. Other variables were taken from medical history records like duration with diabetes since diagnosis, type of diabetes, the presence of complications, and fasting blood sugar during the first diagnosis of diabetic patients of both cases and controls. To control the quality of the data to be collected, the questionnaire was initially prepared in English by language expert, and this was translated into local language (Tigrigna). This questionnaire, prepared in local language, was translated back to English by another language expert to ensure consistency. Data were collected by six nurses (B.Sc.) and two supervisors (M.Sc.). Training was given to data collectors by the principal investigators and supervisors in Aksum Town for two days. A week prior to the actual data collection, the questionnaire was pre-tested on 5% of the total sample size in a hospital not included in the actual data collection (Suhul Hospital). Following the pretest, the actual data were collected, reviewed and checked for completeness and consistency by the supervisor and by the principal investigator daily. Weight (in kilograms) was measured in light clothing and without shoes using calibrated UNICEF Seca Digital Weighing Scale and was checked every six patients by another calibrated UNICEF Seca Digital Weighing Scale [14]. Height was also measured using Stadiometer in centimeter (cm) in an erect position in which the back of the head, shoulder blades, buttocks, and heels make contact with the backboard at a precision [15]. The collected data were manually checked for their completeness and then entered into Epi data version 3.1. The data was analyzed using SPSS version 23. Analysis using bivariable logistic regression model was made to see the association between the explanatory variables and the outcome variable. This was followed by multivariate logistic regression analysis using those variables with P-value ≤0.2 in the bivariable analysis. To check the goodness of fit of the statistical model, the Hosmer-Lemen show test was used. Multicollinearity was assessed by variance inflation factor. All assumptions of binary logistic regression were checked. Odds ratio with 95% CI was used to measure the strength between the dependent and the independent variables. P Value < 0.05 was used to determine the level of statistical significance. This study operationalized the variables as follows.

**Adherence to exercise:** a patient was considered to adhere to exercise when he/she scored at least 50% of the total SDCA [15, 16].

**Adherence to dietary regimen:** a patient was considered to adhere to dietary regimen when he/she scored at least 50% of the total MMS dietary related questions [13, 17].

**Adherence to medication:** a patient was considered to adhere to medication when he/she scores at least 80% of the total Morisky medication scale related questions [17].

**Adherence to blood glucose monitoring:** adherence was recorded when the patients score at least 50% summary of diabetic care blood sugar testing questions [15, 16].

**Good glycemic control:** a good glycemic control was considered when a patient achieved and maintained a mean HbA1c ≤ 7% [18].

**Poor glycemic control:** a poor glycemic control was considered when a patient had HbA1c higher than 7% for adult diabetic patients, and higher than 8% for comorbid, vascular complications, age greater than 60 and history of sever hypoglycemia [18].

## Results

**Socio-demographic characteristics of the respondents:** in this research, a total of 101 DM patients who had hypertension (cases) and 202 DM patients who had no hypertension (controls) were included with a response rate of 100%. 54 (52.9%) cases and 111 (54.9%) controls were male participants. The minimum and maximum age of the DM patients was 18 and 88 years, respectively, with their median age being 51.56±14.92 years. Most of the cases (81.2%) and controls (66.8%) were living in urban areas and the majority of the cases (75.3%) and controls (69.3%) were married. Regarding the educational status, only few of the cases (26.7%) and controls (26.7%) completed primary school. Ethnic-wise, almost all of the cases (99%) and controls (98.5%) were Tigrayans, and a similarly high percentage of cases (85.2%) and controls (88.1%) were Orthodox Christians by religion. Occupation-wise, 32 (31.8%) of the cases and 58 (28.7%) of the controls were private employees Table 1.

**Table 1:** socio-demographic characteristic of diabetes mellitus patients at public hospitals, central zone, Tigray, 2018

Variables	Controls	Cases	Total
**Sex**			
Male	111(54.9%)	54(52.9%)	165(54.5%)
Female	91(45.1%)	47(46.1%)	138(45.5%)
**Age**			
<60 years	155(76.7%)	73(72.3%)	228(75.2%)
60-70 years	31(15.3%)	20(19.8%)	51(16.8%)
>70 years	16(7.9%)	8(7.9%)	24(7.9%)
**Residence**			
Urban	135(66.8%)	82(81.2%)	217(71.6%)
Rural	67(33.2%)	19(18.8%)	86(28.4%)
**Marital status**			
Married	140(69.3%)	76(75.3%)	216(71.3%)
Single	35(17.3%)	4(3.9%)	39(12.9%)
Widowed	7(3.5%)	10(9.9%)	17(5.6%)
Divorced	20(9.9%)	11(10.9%)	31(10.2%)
**Educational level**			
Cannot read and write	52(25.7%)	23(22.8%)	75(24.8%)
Can read and write	21(10.4%)	11(10.9%)	32(26.7%)
Primary school	54(26.7%)	27(26.7%)	81(26.7%)
Secondary school	37(18.5%)	14(13.8%)	51(16.8%)
Colleague and above	38(18.8%)	26(25.8%)	64(21.1%)
**Occupation**			
House wife	48(23.7%)	25(24.7%)	73(24.1)
Governmental employee	52(25.7)	24(23.7%)	76(25.1%)
Private employee	58(28.7%)	32(31.8%)	90(29.7%)
Daily worker	6(2.9%)	2(1%)	8(2.6%)
Farmer	38(18.8%)	18(17.8%)	56(18.5%)
**Ethnicity**			
Tigray	199(98.5%)	100(99%)	299(98.7%)
Amhara	2(1%)	1(1%)	3(1.0%)
Oromo	1(0.5%)	0(0.0%)	1(0.3%)
**Religion**			
Orthodox	178(88.1%)	86(85.2%)	264(87.1%)
Muslim	24(11.9%)	15(14.8%)	39(12.9%)

**Health profile of the respondents:** the mean time of the respondents since diagnosis with DM was 5.5 years (95% CI) with a minimum of 1 year and a maximum of 26 years. More than half of the cases (56.4%) and a third of the controls (34.7%) had a family history of diabetes. Unlike controls (23.3%), the majority of the cases (82.2%) were members of the diabetes association. Only few cases (16.8%) and controls (23.8%) attended education about DM. The majority of the cases (96%) and controls (69.8%) were having type two DM. Most of the cases (90.1%) but only few of the controls (21.3%) had a medically confirmed comorbidity and a comparable magnitude of cases (43.6%) and controls (39.6%) were overweight. The majority of the cases (72.3%) and controls (79.1%) had a glucometer in their home and a similarly high percentage of the cases (81.1%) and controls (73%) were taking oral hypoglycemic medication as part of the treatment of their DM Table 2.

**Table 2:** health profile of the respondents at public hospitals, Central zone, Tigray, 2018

Variable	Controls	Cases	Total
**Duration with DM**			
≤one year	30(14.9%)	3(3%)	33(10.9%)
2-5 year	93(46%)	46(45.5%)	139(45.9%)
≥ six years	79(39.1%)	52(51.5%)	139(45.9%)
**Comorbidity**			
Yes	43(21.3%)	91(90.1%)	134(44.2%)
No	159(78.7%)	10(9.9%)	169(55.8%)
**Current medication you take**			
Insulin	54(26.99%)	19(18.8%)	73(24.1%)
Oral hypoglycemic	146(73%)	82(81.2%)	228(75.2%)
Both	2(0.01%)	0(0)	2(0.7%)
**Family history of DM**			
Yes	70(34.7%)	57(56.4%)	127(41.9%)
No	132(65.3%)	44(43.6%)	176(58.1%)
**Do you attend diabetic education session**			
Yes	48(23.8%)	17(16.8%)	65(21.5%)
No	154(76.2%)	84(83.2%)	238(78.5%)
**Are you Member of DM association**			
Yes	47(23.3%)	81(80.2%)	65(21.5%)
No	155(76.7%)	23(22.8%)	78(25.7%)
**Do you Have glucometer at home**			
Yes	161(79.7%)	73(72.3%)	234(77.2%)
No	41(20.3%)	28(27.7%)	69(22.8%)
**BMI status**			
Under weight	38(18.8%	7(6.9%)	45(14.9%)
Normal	84(41.6%)	50(49.5%)	134(44.2%)
Over weight	80(39.6%)	44(43.6%)	124(40.9%)
**Type DM**			
Type one	61(30.2%)	4(4%)	65(21.5%)
Type two	141(69.8%)	97(96%)	238(78.5%)

**Behavioral factors and respondent's knowledge about diabetic mellitus:** while less than a quarter of cases (23.8%) and most of the controls (66.3%) adhered to their medication, adherence to diet was very low in both cases (20.8%) and controls (10.9%). With regard to adherence to exercise, more controls (78.2%) than cases (61.4%) adhered to exercise. Likewise, unlike the cases (37.6%), most of the controls (84.2%) adhered to blood glucose monitoring. While a substantial number of cases (90.1%) and controls (61.4%) had poor glycemic control, only few of the cases (5%) and controls (4%) were current smokers. In relation to knowledge regarding diabetes mellitus 36.6% cases and 24.3% controls had poor knowledge Table 3. In bivariate analysis, the independent variables that showed an association with the outcome variable were residence, marital status, age, having glucometer at home, attendance of diabetes education, membership of diabetic association, knowledge about DM, family history of DM, duration with DM, type of diabetes, comorbidity, glycemic control, body mass index, adherence to medication, adherence to blood glucose monitoring and adherence to exercise. After considering all assumptions of binary logistic regression, those variables with p- value ≤0.2 in bivariable analysis were entered into multivariable logistic regression. In the multivariable logistic regression analysis, eight variables were found to be determinants of hypertension among diabetic patients at 5% level of significance. Those who had poor glycemic control were significantly associated with hypertension among diabetic patients. The odds of being poor glycemic control in those diabetic patients with hypertension was 22.99 times more (AOR=22.99: at 95% CI (5.92, 89.28) than those diabetic patients with no hypertension.

**Table 3:** behavior factors and knowledge about diabetes mellitus of the respondents at public hospitals, Central zone, Tigray, 2018

Variables	Controls	Cases	Total
**Adherence to diabetic Medication**			
Adhere	134(66.3%)	24(23.8%)	158(52.1%)
Not adhere	68(33.7%)	77(76.2%)	145(47.9%)
**Adherence to diabetic diet**			
Adhere	180(89.1%)	80(79.2%)	260(85.8%)
Not adhere	22(10.9%)	21(20.8%)	43(14.2%)
**Adherence to diabetic exercise**			
Adhere	158(78.2%)	62(61.4%)	220(72.6%)
Not adhere	44(21.8%)	39(38.6%)	83(27.4%)
**Adherence to blood glucose**			
Adhere	170(84.2%)	38(37.6%)	208(68.6%)
Not adhere	32(15.8%)	63(62.4%)	95(31.4%)
**Current smoker**			
Yes	4(2%)	5(5%)	9(3%)
No	198(98%)	96(95%)	294(97%)
**Past smoker**			
Yes	5(2.5%)	3(3%)	8(2.6%)
No	197(97.5%)	98(97%)	295(97.4%)
**Glycemic control**			
Good glycemic	78(38.6%)	10(9.9%)	88(29%)
Poor glycemic	124(61.4%)	91(90.1%)	215(71%)
**Knowledge about DM**			
Good knowledge	153(75.7%)	64(63.4%)	217(71.6%)
Poor knowledge	49(24.3%)	37(36.6%)	86(28.4%)

The respondents who had not adhered to self-monitoring of blood glucose (SMBG) level were 6. (62 times more likely to have hypertension than those who had adhered to SMBG AOR=6.62: 95% CI, (3.16, 13, 86). Likewise, non-adherence to exercise among diabetic patients made patients to be 5.47 more likely to develop hypertension than those with good adherence (AOR=5.47 at 95CI, (2.35, 12.75). Moreover, respondents who were overweight diabetic patients were strongly associated with hypertension than with underweight respondents. The odds of hypertension in overweight DM patients was 4.84 times more than those underweight respondents (AOR=4.84 at 95CI (1.42, 16.51). Non-adherence to diabetic diet also showed association to be associated with the development of hypertension among diabetic patients. The power of non-adherence to diet was 9.7 times highly associated with the outcome variable than their counterparts (AOR=9.70 at 95 CI (3.34, 28.22). Non-adherence to diabetic medications showed a relationship with hypertension among diabetic patients. The odd of developing hypertension was 4.66 times strongly associated with those who were not adhering to their medication than their counterparts (AOR=4.66 at 95CI (2.22, 9.79). Those respondents who stayed with DM for a long period of time showed association with hypertension among DM patients than those who stayed with DM for short duration of time (AOR=9.44 (2.14, 41.75) and 8.52(1.97, 36.84). Lastly, the DM patients who did not attend diabetic education session showed association with hypertension than those who did not attend diabetic education session (AOR=2.61, 95 CI (1.12, 6.11) (Table 4).

**Table 4:** multiple logistic regression analysis for association with hypertension among diabetes mellitus patients in public hospitals, central zone, Tigray 2018

Variable	Controls	Cases	COR [95%CI]	AOR [95%CI]	p-value
**Duration with diabetics**					
≤one year	30(14.9%)	3(3%)	1	1	
2-5 year	93(46%)	46(45.5%)	4.95[1.43,17.06]	9.44	0.03*
≥ six years	79(39.1%)	52(51.5%)	6.58[1.91,22.68]	8.52	0.04*
**Attend diabetic education sessions**					
Yes	48(23.8%)	17(16.8%)	1	1	
No	154(76.2%)	84(83.2%)	1.54[0.83,2.84]	2.61	0.027*
**BMI status**					
Under weight	38(18.8%	7(6.9%)	1	1	
Normal	84(41.6%)	50(49.5%)		0.11	0.111*
Over weight	80(39.6%)	44(43.6%)	2.98[1.23,7.24]	4.84	0.012*
**Adherence to Medication**					
Adhere	134(66.3%)	24(23.8%)	1	1	
Not adhere	68(33.7%)	77(76.2%)	6.32[3.67,10.88]	4.66	0.000*
**Adherence to Diet**					
Adhere	180(89.1%)	80(79.2%)	1	1	
Not adhere	22(10.9%)	21(20.8%)	2.15[1.12,4.13]	9.70	0.000*
**Adherence to diabetic Exercise**					
Adhere	158(78.2%)	62(61.4%)	1	1	
Not adhere	44(21.8%)	39(38.6%)	2.26[1.34,3.80]	5.47	0.000*
**Adherence to blood glucose**					
Adhere	170(84.2%)	38(37.6%)	1	1	
Not adhere	32(15.8%)	63(62.4%)	9.09[5.23,15.84]	6.62	0.000*
**Glycemic control**					
Good glycemic	78(38.6%)	10(9.9%)	1	1	
Poor glycemic	124(61.4%)	91(90.1%)	5.72[2.81,11.66]	22.99	0.000*

* Shows those variables significantly associated with the outcome variable at p-value <0.05

## Discussion

The study provides information about the determinants of hypertension among DM patients in public hospitals in the Central Zone of Tigray, Ethiopia in 2018. Study subjects with poor glycemic control were significantly associated with hypertension among DM patients. The odds of hypertension in DM patients with poor glycemic control was 23 times more than those with diabetic patients with good glycemic control. This study was in line with a cross-sectional study conducted in India and Japan [19-21]. This association might be because of reduced cellular response to insulin, which in turn prompts an increased tone of the smooth muscles of blood vessels. Additional contributing factors could be increased tissue inflammation and production of reactive oxygen species which might result in endothelial dysfunction, and increased tissue renin-angiotensin-aldosterone system and stimulation of sympathetic nervous system which might result in constriction of smooth muscle activity. All these changes and biological mechanisms might have been involved in this complex pathophysiology of hypertension in diabetes [22]. Respondents who did not adhered to a regular blood glucose monitoring were 6.62 times more likely to be associated with hypertension compared to their counterparts. This study was similar with a cohort study conducted in Northern California and South Yorkshire in 2001 and 2007 [23]. This might be related to prior self-care practice and glucose level control; a regular measuring of glucose level and performing recommended self-care actions like healthy diet, exercise and diabetic education play key role in glycemic control. More frequent self-monitoring of blood glucose level was associated with clinically and statistically better glycemic control that prevent further complication of CVD such as hypertension. These findings were supported by the clinical recommendations suggested by the American Diabetes Association. Non-adherence to exercise showed significant association with hypertension among diabetic patients than those who adhere to exercise. Patients who were non-adherent to exercise were 5.47 times more likely to be predisposed to hypertension compared to their counterparts. This result is supported by studies conducted in different region of the continents [24-27]. This association might be due to the fact that exercise can appreciably reduce body weight, systemic vascular resistance, plasma norepinephrine, waist circumference, body fat, and insulin resistance. On the other hand, exercise can increase high-density lipoprotein and cell insulin sensitivity which results in good glycemic control.

Overweight diabetic patients were strongly associated with hypertension compared with the underweight patients. The odds of having hypertension among overweight patients was 4.84 times more when compared with the underweight patients. This finding is supported by different studies conducted in different countries [28-32]. This relationship between weight and hypertension could be related to the high risk of overweight patients for developing arterial stiffness and endothelial dysfunction which increases blood pressure by rising renal tubular reabsorption, impairing pressure natriuretic, and causing volume expansion due to activation of the sympathetic nervous system and renin-angiotensin-aldosterone system and by physical compression of the kidneys. Diabetic patients, who did not adhere to diabetic diet, showed association with hypertension. The odds of hypertension in those who were not adhering to diet was 9.7 times more than those who were adhering to diet. This might be because high fiber intake is associated with lower serum cholesterol concentrations, lower risk of coronary heart disease, reduced blood pressure, enhanced weight control, better glycemic control, and reduced risk of cardiovascular diseases [31, 33]. This study was also supported by another controlled trial study conducted in 2003 and 2004 [34, 35]. Non-adherence to medication also showed association with hypertension among diabetic patients than their counterparts. The odds of developing hypertension were 4.66 times more probable for patients who were adhered to their medication compared to those who did not do. This might be true because adherence to DM medications is key measure not only to keep an optimal blood sugar level but also decreases the likelihood of developing hypertension among DM patients [36-38]. This study showed that longer duration with DM was found to be a contributing factor for hypertension. A long period of time since diagnosis was 8.52 times more predisposing to hypertension than those with short period of time. The possible explanation could be that the natural processes related to duration, such as autonomous imbalance and blood vessel stiffening get increased with time, which in turn are highly related to the development of hypertension. The DM patients who did not attend diabetic education session had significant association with hypertension than their counterparts [39-41]. The probable reasoning could be because the diabetic education session helps practicing the behavioral factors such as glucose control, adherence to exercise, adherence to SMBG, adherence to diet and adherence to medication [41-44]. Patient education is a key factor for optimal therapy of diabetes and other chronic conditions. When an education is offered effectively, it leads for better self-care and improved adherence [45].

## Conclusion

This research concludes that long duration with diabetes since diagnosis, non-attendance of diabetic self- management education, poor glycemic control, and non-adherence to diabetic medication, diet, exercise and self-monitoring blood glucose were found to be the determinants of hypertension among diabetic patients.

### What is known about this topic

- The prevalence of hypertension among diabetic patients in Africa is a major public health problem;
- Hypertension has become the commonest cause of cardiovascular diseases, renal and stroke and another disease on the continent;
- If not effective dealt, by 2020, three-fourths of all deaths in Africa will be attributable to hypertension.

### What this study adds

- Long duration with diabetes since diagnosis, not attend diabetic education sessions, and not-adherent to diabetic medication, diet, exercise and blood glucose were found to be the determinants of hypertension among diabetic patients;
- Health care personnel should improve the patients practice to the domains of diabetic management by strengthening information, education and communication programs;
- Another research should be carried out to investigate the determinants of hypertension among diabetic patients in a broader social context and in a larger sample size.

## Competing interests

The authors declare no competing interests.
